# The Effects of Balloon Occlusion of the Aorta on Cerebral Blood Flow, Intracranial Pressure, and Brain Tissue Oxygen Tension in a Rodent Model of Penetrating Ballistic-Like Brain Injury

**DOI:** 10.3389/fneur.2019.01309

**Published:** 2019-12-18

**Authors:** Zachary S. Bailey, Katherine Cardiff, Xiaofang Yang, Janice Gilsdorf, Deborah Shear, Todd E. Rasmussen, Lai Yee Leung

**Affiliations:** ^1^Brain Trauma Neuroprotection, Center for Military Psychiatry and Neuroscience, Walter Reed Army Institute of Research, Silver Spring, MD, United States; ^2^Department of Surgery, Uniformed Services University of the Health Sciences, Bethesda, MD, United States

**Keywords:** resuscitative endovascular balloon occlusion of the aorta, penetrating brain injury, intracranial pressure, cerebral blood flow, brain tissue oxygen tension

## Abstract

Trauma is among the leading causes of death in the United States. Technological advancements have led to the development of resuscitative endovascular balloon occlusion of the aorta (REBOA) which offers a pre-hospital option to non-compressible hemorrhage control. Due to the prevalence of concomitant traumatic brain injury (TBI), an understanding of the effects of REBOA on cerebral physiology is critical. To further this understanding, we employed a rat model of penetrating ballistic-like brain injury (PBBI). PBBI produced an injury pattern within the right frontal cortex and striatum that replicates the pathology from a penetrating ballistic round. Aortic occlusion was initiated 30 min post-PBBI and maintained continuously (cAO) or intermittently (iAO) for 30 min. Continuous measurements of mean arterial pressure (MAP), intracranial pressure (ICP), cerebral blood flow (CBF), and brain tissue oxygen tension (PbtO_2_) were recorded during, and for 60 min following occlusion. PBBI increased ICP and decreased CBF and PbtO_2_. The arterial balloon catheter effectively occluded the descending aorta which augmented MAP in the carotid artery. Despite this, CBF levels were not changed by aortic occlusion. iAO caused sustained adverse effects to ICP and PbtO_2_ while cAO demonstrated no adverse effects on either. Temporary increases in PbtO_2_ were observed during occlusion, along with restoration of sham levels of ICP for the remainder of the recordings. These results suggest that iAO may lead to prolonged cerebral hypertension following PBBI. Following cAO, ICP, and PbtO_2_ levels were temporarily improved. This information warrants further investigation using TBI-polytrauma model and provides foundational knowledge surrounding the non-hemorrhage applications of REBOA including neurogenic shock and stroke.

## Introduction

Traumatic hemorrhage is the leading cause of potentially preventable death in both civilian and military populations (1, 2). Medical response to a traumatic event is limited by the lack of technology to control non-compressible hemorrhage which has prompted the development and implementation of resuscitative endovascular balloon occlusion of the aorta (REBOA) (3–7). The REBOA procedure includes the placement of a balloon catheter into the descending aorta to temporarily occlude distal blood flow and augment cerebral and myocardial perfusion. The procedure is more feasible in a pre-hospital setting, technically easier for non-trauma surgeons, and is less invasive than the traditional resuscitative thoracotomy with aortic cross clamping. Unfortunately, traumatic events often involve complex injury patterns and patients are often faced with polytrauma. Neurologic injury, including traumatic brain injury (TBI), is among the most common factor in polytrauma patients and can worsen outcome (8, 9). It is important to understand the effects of REBOA on concomitant TBI because the significant hemodynamic shift induced by the occlusion likely impacts the cerebral perfusion, intracranial pressure (ICP), and oxygen delivery following injury.

To address this knowledge gap, Shackford et al. (10) used a porcine model of cryogenic brain lesion and aortic cross clamping to show no adverse effects on ICP, cerebral blood flow (CBF), or water content. These results were replicated with a controlled cortical impact model and expanded to show no worsening of hemorrhage progression in adult swine (11, 12). However, Williams et al. (12) demonstrated that TBI may worsen the effects of hemorrhagic shock. Prior to translating these results to human patients, the anatomical differences of the swine cerebrovasculature must be considered. The most notable feature is the presence of a carotid rete mirabile which is not found in humans (13–15). This structure lies on route of the main arterial blood supply to the brain and has been shown to provide flow-dampening effects due to its resistance to blood flow (13, 16). Therefore, the blood flow changes induced by aortic occlusion in the porcine models may be dampened before reaching the brain and may not adequately represent the human cerebrovasculature system.

Aortic occlusion is not intended as a therapy for TBI, especially in normotensive patients. Rather, the goal of this study is to provide foundational knowledge surrounding the relationship between aortic occlusion and cerebral physiology following severe, penetrating TBI. In order to isolate this relationship, we employed a rat model of penetrating ballistic-like brain injury (PBBI) which provides an optimal testing platform for several reasons: (1) severe intracranial hemorrhage is a prominent feature of the injury; (2) cerebral blood flow changes can be studied in the absence of a carotid rete mirable; (3) in contrast to blunt TBI, penetrating TBI is visible so actionable information is available for making treatment decisions; and (4) the isolated brain injury will create an opportunity to better understand the relationship between the injured brain and aortic occlusion before being incorporated into a polytrauma model. In this study, continuous (cAO) or intermittent (iAO) aortic occlusion will be induced by an embolectomy balloon catheter following PBBI. The iAO procedure was adapted from Morrison et al. (17) who sought to minimize the ischemia, inflammatory, and metabolic burden following occlusion by incorporating 1 min periods of balloon deflation (17). This method of occlusion was important to test in the presence of brain injury because the repeated blood flow changes may elicit different effects on the brain recovery. We hypothesize that both cAO and iAO will increase cerebral perfusion which will augment brain tissue oxygen tension and intracranial pressure following PBBI. This information will provide groundwork to begin to more closely understanding cerebral physiological responses in polytrauma models and will be important to facilitating an efficient translation of REBOA protocols to patients with concomitant, severe TBI. Importantly, these results will also yield foundational knowledge surrounding the non-hemorrhage clinical applications of REBOA, including neurogenic shock (18), cerebral ischemia (19), and non-traumatic cardiac arrest (20).

## Materials and Methods

### Experimental Design

All procedures involving animal use were reviewed and approved by the Institutional Animal Care and Use Committee of Walter Reed Army Institute of Research (Silver Spring, MD). Research was conducted in compliance with the animal welfare act, Guide for the Care and Use of Laboratory Animals (NRC Publication, 2011 edition), and other federal statutes and regulations. Animals were housed individually under a 12 h light/dark cycle in an AAALAC International accredited facility on a 5-pellet per day diet of ProLab Irradiated 3000 Rodent Diet (5P76, LabDiet, St. Louis, MO) with water provided *ad libitum*.

**Figure 1:**
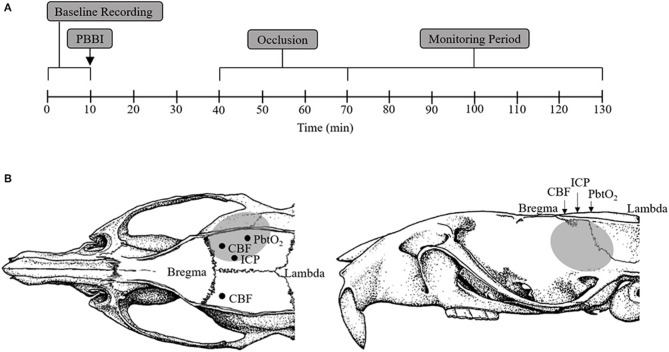
Schematic representation of sensor probe locations and experimental procedure timeline. **(A)** Schematic representation of the experimental timeline including baseline (1–10 min), PBBI (at 10 min), aortic occlusion (40–70 min), and monitoring period (70–130 min). **(B)** Stereotaxic positions of ICP (AP: −2.5 mm, ML: +1.5 mm), PbtO_2_ (AP: −4 mm, ML: +4 mm), and CBF (AP: −1 mm, ML: ±3 mm) sensor probes. The gray region represents the lesion core and penumbra.

Adult male Sprague-Dawley rats (480–680 g; Charles River Labs, Raleigh, VA) were randomly divided into the following four treatment groups (*n* = 10/group): sham+cAO, PBBI+cAO, sham+iAO, PBBI+iAO. All rats were instrumented for continuous monitoring of femoral/carotid mean arterial pressure (MAP), breathing rate, heart rate, ipsilateral/contralateral CBF, ICP, and brain tissue oxygen tension (PbtO_2_) during all procedures. After a 10 min baseline recording period, rats were either subjected to a right frontal cortex PBBI or a craniotomy without injury (sham procedure). Occlusion of the descending aorta was initiated 30 min after the PBBI or sham procedures. Little is understood about acceptable occlusion durations, however total occlusion times of 40 min have been considered safe, after which risks may outweigh the benefits (21, 22). Therefore, the aorta was occluded for a total of 30 min for both cAO and iAO. Following occlusion, physiological recordings were continued for 60 min. At completion of the monitoring phase and while under anesthesia, animals were euthanized by CO_2_ inhalation according to the guidelines of the 2013 American Veterinary Medical Association Guidelines on Euthanasia. Figure 1A provides a schematic representation of these experimental procedures.

### Surgical Preparation

All surgical procedures were carried out under aseptic technique. Animals were induced with 3–4% isoflurane delivered in air/oxygen mixture (FiO_2_ = 0.26) and maintained at 2.5% throughout the surgical preparations. All surgeries were carried out with careful monitoring of vital signs. Core body temperature was maintained at 37°C using a homeothermic heating system (Harvard Apparatus, Holliston, MA). An external pulse transducer (ADInstruments, INC., Colorado Springs, CO) was positioned in contact with the chest wall to monitor breathing. Electrocardiogram signal was collected using needle electrodes placed in each forelimb and the left hindlimb, and amplified using the Animal Bio Amp (ADInstruments, INC., Colorado Springs, CO). Heart rate was calculated from the measured electrocardiogram signal. Distal and proximal blood pressure transducers (Harvard Apparatus, MA) were used to record MAP via a left femoral artery and a right common carotid catheter, respectively.

Following catheterization, animals were fixed on a stereotaxic frame. A cranial window (AP: +4.5 mm, ML: +2 mm from Bregma) was trephined for PBBI procedures. Skull trephination was also performed for the insertion and placement of ICP (AP: −2.5 mm, ML: +1.5 mm), PbtO_2_ (AP: −4 mm, ML: +4 mm), and CBF (AP: −1 mm, ML: ±3 mm) probes (Figure 1B). ICP and PbtO_2_ were measured in the ipsilateral cerebral cortex while CBF was measured in both the ipsilateral and contralateral cerebral cortices. ICP, PbtO_2_, and CBF measurements were made using the Codman ICP Express (Johnson & Johnson, Raynham, MA), Licox brain tissue oxygen probes (Integra Neuroscience, Plainsboro, NJ), and laser Doppler flowmetry (Moor Instruments, Wilmington, DE), respectively. Following placement and stabilization of all the required probes, the isoflurane was reduced to 1.5% and a 10 min baseline recording period was initiated. All physiological parameters were recorded simultaneously using PowerLabs data acquisition system (ADInstruments, Inc., Colorado Springs, CO) and analyzed using LabChart v7 (ADInstruments, Inc., Colorado Springs, CO).

### PBBI Procedures

Following the 10 min baseline data collection, unilateral (right) PBBI was induced using a simulated ballistic injury device (Mitre Corp., McLean, VA) with a specially designed stainless steel probe (Popper & Sons Inc., New Hyde Park, NY). The probe was manually inserted 12 mm into the right frontal cortex through the cranial window at an angle of 50° anterior from the vertical axis and 25° counter-clockwise from the anterior-posterior axis. The elastic tubing on the probe was inflated by a rapid water pressure pulse which formed an elliptical balloon. The balloon was calibrated to 10% of the total rat brain volume causing an intracerebral cavity (Figure 1B). The probe was retracted, and the cranial opening was sealed with bone wax.

### Aortic Occlusion Procedures

Prior to injury, a 2F Fogarty arterial embolectomy catheter (Edwards LifeSciences) was inserted into the right femoral artery and advanced into the abdominal aorta between the lowest renal artery and the aortic bifurcation. The optimal insertion length of the catheter was determined to be ~8 cm by post-mortem dissection. The balloon was inflated with normal saline to induce aortic occlusion beginning 30 min after PBBI. For cAO, the occlusion was held for 30 min. To induce iAO, the balloon was deflated for 1 min every 10 min while maintaining a total occlusion time of 30 min.

### Statistical Analysis

The sample sizes for all groups was determined by power analysis (power = 0.8; alpha = 0.05) using G^*^Power 3 based on means and standard deviations of our own previous data. Unless otherwise noted, all data is expressed as mean ± standard error of the mean (SEM). For all continuous physiological parameters, the data was analyzed as 2 min averages taken at 2 min intervals across the entire recording period. In order to test our hypothesis, statistical comparisons were performed for direct evaluation of the effects of PBBI in the presence of either cAO or iAO. Therefore, PBBI groups were directly compared to their sham counterparts. Since the medical decision of implementing cAO or iAO is dependent on environmental factors, specific details of the injury, and time to medical evacuation, direct comparisons between cAO and iAO were beyond the scope of this work. Significant differences between PBBI and sham groups were assessed over time using a 2-way repeated measures analysis of variance. When necessary, *post-hoc* analysis was performed using Fisher's Least Significant Difference test. *p* < 0.05 is considered statistically significant.

## Results

**Figure 2:**
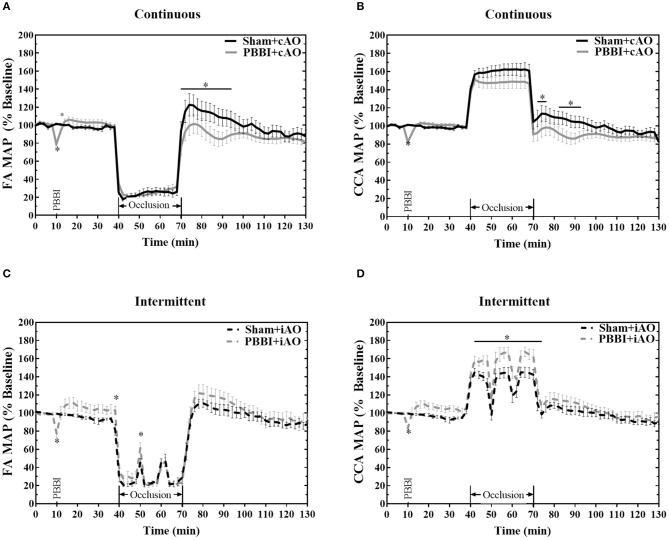
Hemodynamic changes induced by aortic occlusion. Aortic occlusion elicited a dramatic shift in hemodynamics characterized by decreased femoral artery **(A,C)** and increased carotid artery **(B,D)** MAP. Data expressed as mean ± SEM. *p < 0.05 compared to sham.

The ability of the aortic occlusion procedure to effectively regulate the systemic hemodynamics in both sham and PBBI animals is demonstrated in Figure 2. Inflation of the endovascular balloon (at 40 min) triggered a hemodynamic shift and occluded blood flow through the descending aorta. The distal MAP measured at the femoral artery was significantly reduced but maintained at ~20 mmHg which likely resulted from collateral circulation during full occlusion.

The reduced femoral artery MAP was accompanied by a simultaneous increase in carotid artery MAP which is indicative of augmented central aortic pressure (Figures 2B,D). During occlusion, the MAP in the carotid artery increased by an average of 53.4 ± 0.4 mmHg over baseline values, reaching an average value of 153.6 ± 0.3 mmHg. A similar increase of 55 mmHg has been observed in clinical studies of REBOA intervention (23, 24). During the iAO procedures, carotid MAP decreased toward physiologically normal levels during periods of balloon deflation (Figure 2D).

PBBI induced an immediate decrease of MAP followed by a slight, but not significant, increase from the baseline values. No significant changes were observed during cAO, but PBBI+cAO animals showed a temporary decrease in MAP compared to sham+cAO when aortic blood flow was restored (*p* < 0.05). In contrast, PBBI+iAO showed increased carotid MAP during occlusion compared to sham+iAO (*p* < 0.05), and no changes following occlusion. Interestingly, gradual decreases in MAP were observed during the 60 min post-occlusion monitoring period in all treatment groups.

**Figure 3:**
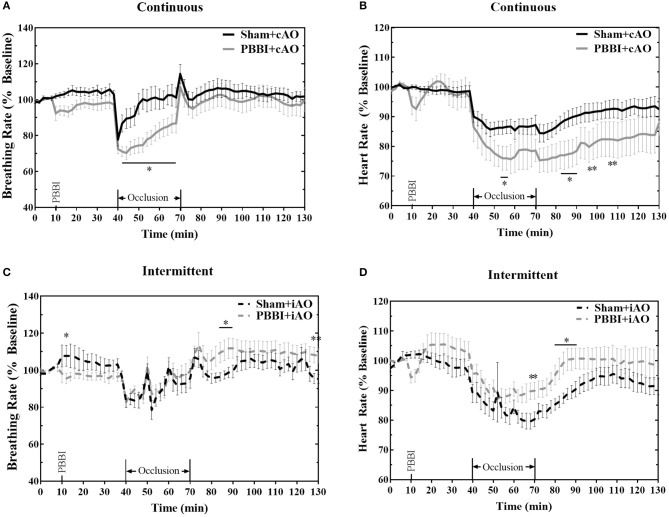
Aortic occlusion leads to temporary changes in breathing rate and heart rate. REBOA triggered a decrease in breathing rate **(A,C)** and heart rate **(B,D)** during occlusion which recovered in the post-occlusion monitoring period. Occlusion effects were similar between both PBBI and sham animals. Data expressed as mean ± SEM. *p < 0.05 compared to sham. The ** symbols apply to consecutive time points in the figure.

PBBI led to trending decreases in breathing and heart rate, but these changes were not statistically significant from sham (Figure 3). Both cAO and iAO elicited a profound decrease on breathing and heart rates in all treatment groups. Overall, the breathing rate decreased by an average of 12.2 ± 0.8 breaths per minute within the first 2 min of occlusion. After the initial decline, the breathing rate gradually approached baseline levels over the duration of occlusion. PBBI+cAO animals showed a significant decrease in breathing rate compared to sham+cAO during occlusion (*p* < 0.05; Figure 3A). These changes were transient as no differences were observed after occlusion. PBBI+iAO animals showed no differences during occlusion but periods of hyperventilation were observed during the post-occlusion monitoring period compared to sham+iAO (Figure 3C).

Heart rate was also decreased following the initiation of aortic occlusion. PBBI+cAO caused decreased heart rate compared to sham+cAO animals (*p* < 0.05; Figure 3B). The significant reduction in heart rate was also observed during the early stages of the post-occlusion monitoring period, but subsided by the end of the monitoring period. Alternatively, PBBI+iAO led to increased heart rate compared to sham+iAO during the end of occlusion and early stages after blood flow was restored (*p* < 0.05; Figure 3D).

**Figure 4:**
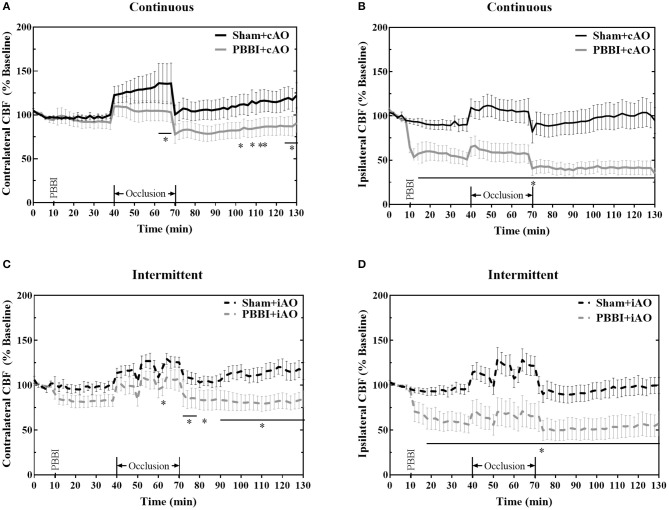
Aortic occlusion is unable to mitigate cerebral hypoperfusion following injury. PBBI induces a significant reduction in ipsilateral CBF **(B,D)** but not contralateral CBF **(A,C)**. Occlusion augments CBF but is not sufficient to restore sham levels. CBF is decreased following PBBI in both hemispheres during the post-occlusion monitoring period. Data expressed as mean ± SEM. *p < 0.05 compared to sham. The ** symbols apply to consecutive time points in the figure.

No significant changes in CBF were observed following injury on the contralateral side (Figures 4A,C), but PBBI induced a dramatic decrease in CBF on the ipsilateral side in both injury groups (Figures 4B,D). Aortic occlusion increased CBF in each treatment group, but the increase was more prominent in sham animals. To more clearly show the occlusion effects on CBF, measurements during the occlusion were normalized to measurements immediately preceding the occlusion (post-PBBI) and are shown in Table 1. Ipsilateral CBF increased by 17.99 ± 0.68 mmHg and 23.99 ± 2.27 mmHg in sham+cAO and sham+iAO groups, respectively. These effects were not observed following PBBI as CBF increased by only 5.11 ± 1.30 and 13.58 ± 3.30 mmHg in PBBI+cAO and PBBI+iAO groups, respectively.

**Table 1:** Aortic occlusion induced CBF changes were reduced in PBBI animals.

	**Ipsilateral**	**Contralateral**
Sham + cAO	17.99 ± 0.68	33.34 ± 1.24
PBBI + cAO	5.11 ± 1.30	12.72 ± 0.65
Sham + iAO	23.99 ± 2.27	47.77 ± 3.90
PBBI + iAO	13.58 ± 3.30	42.13 ± 2.51

*The occlusion-induced changes in CBF are decreased in the injured brain compared to their sham equivalents. Data expressed as the average percentage increase (mean ± SEM) in CBF values from post-PBBI (Time = 10–40 min) to post-occlusion (Time = 40–70 min)*.

Due to the dramatic reduction of CBF in PBBI animals, aortic occlusion was unsuccessful in restoring baseline values of CBF in the injured brain (Figure 4). On the ipsilateral side, the PBBI-induced reduction in CBF persisted through the post-occlusion monitoring period in both PBBI+cAO (Figure 4B) and PBBI+iAO groups (Figure 4D). PBBI+cAO and PBBI+iAO groups both showed decreased contralateral CBF during the post-occlusion monitoring period compared to their respective sham groups (*p* < 0.05). These results suggest that the efficacy of augmenting CBF with either cAO or iAO may be limited.

**Figure 5:**
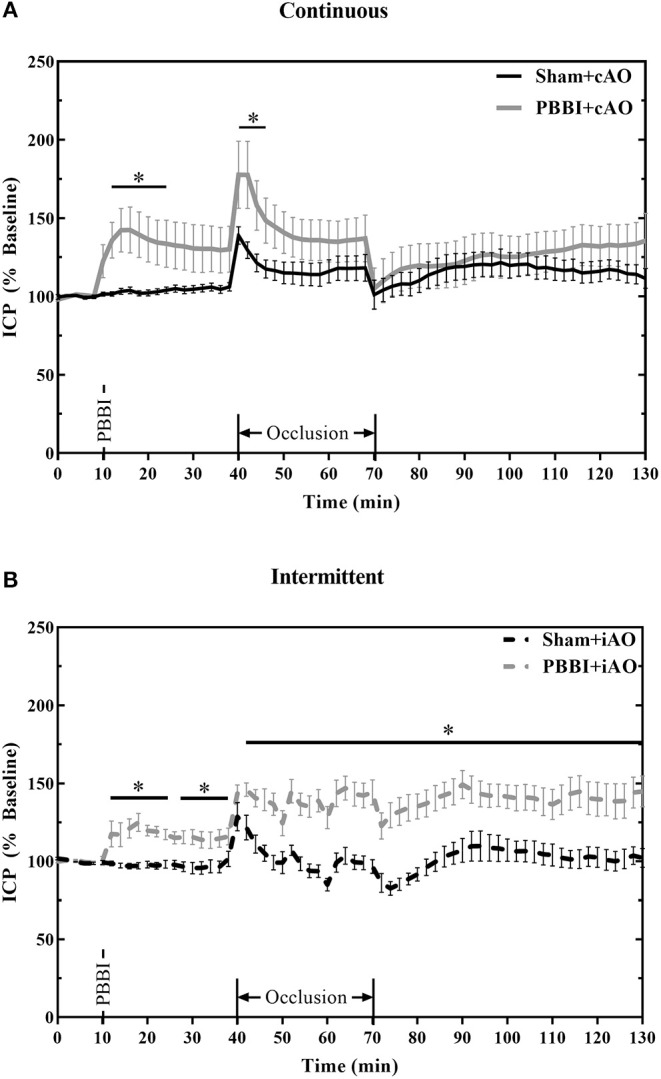
cAO and iAO have differential effects of ICP following PBBI. PBBI increases ICP which was mitigated by cAO **(A)** but not iAO **(B)**. Data expressed as mean ± SEM. *p < 0.5 compared to sham.

Figure 5 summarizes the dynamic ICP responses to both PBBI and aortic occlusion procedures. PBBI significantly increased ICP values compared to sham values for both cAO and iAO groups (*p* < 0.05). cAO caused an abrupt increase in ICP in both sham and PBBI animals (Figure 5A). The initial rise in ICP was followed by a gradual decrease over the duration of aortic occlusion. The PBBI+cAO group demonstrated significantly increased ICP values compared to the sham+cAO animals within the first 10 min of occlusion (*p* < 0.05). Both groups exhibited decreasing trends over the course of occlusion and no significant change was observed over the final 20 min of occlusion or throughout the monitoring period. However, it should be noted that the ICP of the PBBI+cAO group increased gradually during the monitoring period while the sham+cAO group showed little change.

The intermittent occlusion regimen yielded a different ICP response (Figure 5B). Both sham and PBBI animals demonstrated an abrupt rise in ICP within the first few minutes of occlusion. Over the remaining duration of occlusion, sham+iAO animals showed a decreasing trend in ICP whereas the ICP in the PBBI+iAO animals remained significantly elevated (*p* < 0.05). This change was maintained throughout the post-occlusion monitoring period which suggests that iAO may exacerbate the PBBI-induced intracranial hypertension.

**Figure 6:**
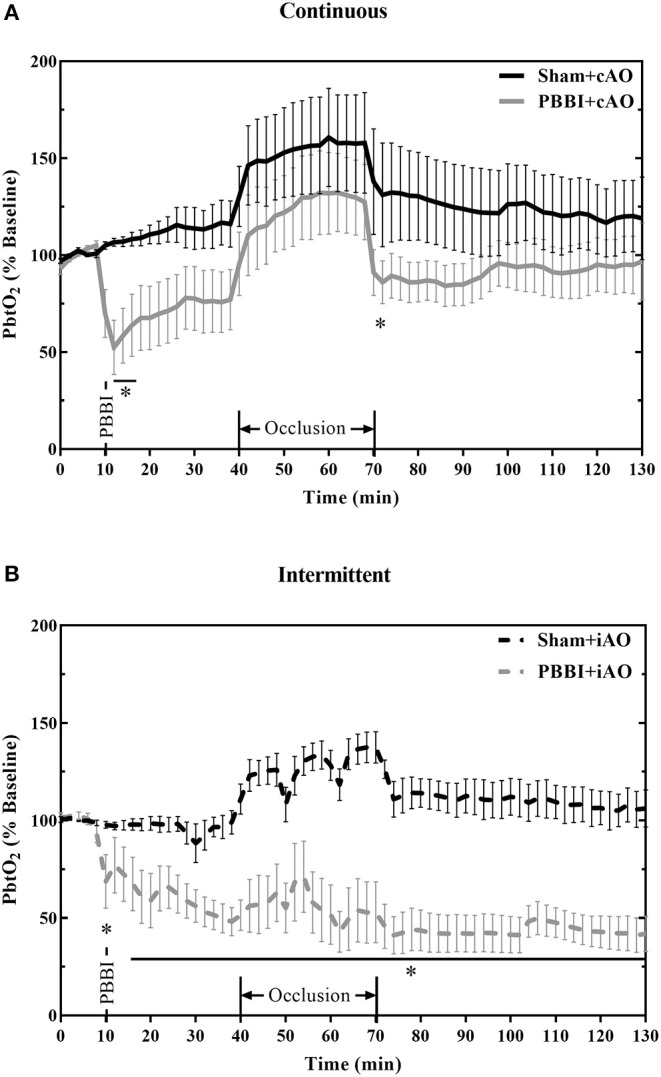
cAO causes temporary augmentation of PbtO_2_ levels during occlusion. PbtO_2_ decreases following PBBI and is increased during cAO **(A)**, but not iAO **(B)**. The effects are not sustained following the completion of aortic occlusion. Data expressed as mean ± SEM. *p < 0.05 compared to sham.

The effects of aortic occlusion on PbtO_2_ levels following PBBI are shown in Figure 6. In cAO and iAO groups, PBBI led to a significant decrease in PbtO_2_ levels compared to their respective shams (*p* < 0.05). Within the first 30 min following injury, PbtO_2_ levels decreased by 30.2 ± 2.1% from baseline values. cAO increased PbtO_2_ levels in both sham and PBBI animals and there was no statistical difference during occlusion (Figure 6A). The observed PbtO_2_ levels were greater than those previously published in a PBBI-only control (25). The cAO effect on PbtO_2_ levels was transient and last only during the occlusion. There was a brief but significant decrease in PbtO_2_ levels in PBBI animals following balloon deflation (p <0.05). During the post-occlusion monitoring period, PBBI+cAO PbtO_2_ levels were not statistically different from sham animals but were similar to those previously reported in the PBBI model (25).

Intermittent occlusion proved to be less successful in augmenting PbtO_2_ during occlusion. Sham+iAO animals demonstrated an increase in PbtO_2_ during occlusion but this change was not detected in the PBBI+iAO animals (Figure 6B). During the post-occlusion monitoring period, the PBBI+iAO group maintained a decreasing trend and remained significantly below sham+iAO levels (*p* < 0.05). The post-occlusion PbtO_2_ levels were also decreased from previously reported PbtO_2_ values in the PBBI model (25).

## Discussion

REBOA has recently gained much attention as it provides a less-invasive and more feasible option toward non-compressible hemorrhage control over traditional methods. However, hemorrhage often occurs with concomitant TBI so understanding the effects of aortic occlusion following TBI will be critical to maintaining vital physiology. The goal of this study was to isolate the relationship between TBI and aortic occlusion to provide basic knowledge of how aortic occlusion may alter acute cerebral physiology but it is important to note that ultimately aortic occlusion is not intended for the normotensive condition or as an isolated TBI therapy. The heart is among the most directly affected organs during periods of aortic occlusion because of the reduced vascular volume and increased cardiac perfusion. The results shown here (Figure 3), along with those reported by others (26, 27), demonstrate the acute inhibitory effects of aortic occlusion on heart rate and breathing rate. Both breathing rate and heart rate were further decreased when cAO occurred following PBBI. In contrast, PBBI+iAO showed little change on breathing rate but slight increases in heart rate compared to sham+iAO. Theoretically, these changes may be influenced by baroreceptor response and brainstem pathology. Previous studies have established a mechanistic role for baroreceptors in the decreased breathing rate and heart rate following aortic occlusion (26, 27). Other studies have demonstrated the vulnerability of baroreceptors to states of prolonged pressure disturbances (28–30). The sudden and dramatic increases in common carotid artery MAP induced by aortic occlusion may elicit a phenomenon previously described as baroreceptor resetting (28). Baroreceptor resetting can be initiated by rapid changes in pressure. When applied for short periods of time (<20 min), the baroreceptors threshold is shifted temporarily with no change in sensitivity (29). Alternatively, when this pressure is applied for longer, the threshold is shifted, the sensitivity is reduced, and the changes are no longer reversible (30). Baroreceptor axons project to the brainstem where parasympathetic activation can regulate breathing rate and heart rate. The PBBI model employed here is a unilateral, frontal injury but has been shown to include secondary injury patterns, including metabolic impairment and neurodegeneration, in remote areas from the injury core (31, 32). This pathology may further complicate the regulation of breathing rate and heart rate.

The mechanical insult in the PBBI model induces extensive cerebrovascular damage that includes blood brain barrier compromise and extravasation of blood components, contributing to the acute and chronic injury pathology (32–34). This leakage causes intracerebral hemorrhage and vasogenic edema formation that can persist for up to 7 days and contribute to ICP aberrations (32–36). Increased ICP is commonly observed following TBI and can often be a useful predictor of neurological outcome (37–39). As a result, acute care and management of TBI often focuses on mitigating the pathological changes in ICP (40, 41). At the beginning of occlusion, all groups saw rapid ICP increases which present a clinical challenge, especially in the pre-hospital environment. To ameliorate this observation in subsequent studies, a partial occlusion or slow inflation of the aortic balloon may be used to avoid rapid changes to ICP. Throughout the duration of occlusion, PBBI+cAO decreased the intracranial hypertension compared to sham+cAO but this was not observed between the PBBI and sham groups following iAO. The decreasing ICP of the PBBI+cAO group during occlusion may be indicative of ongoing blood pressure autoregulatory processes. These innate mechanisms maintain adequate and stable perfusion of the brain by modulating cerebrovasculature resistance in response pressure changes. PBBI+iAO lead to increasing ICP during occlusion which was stabilized and maintained throughout the post-occlusion monitoring period and may provide evidence of compromised pressure autoregulatory mechanisms. Compromised pressure autoregulatory functions have been observed previously following PBBI, but may be exacerbated by the repeated arterial stretch that was induced by the iAO. The ability of cyclic loading to effect artery fatigue and compliance has been observed previously (42, 43). Combined with injury effects to the vasculature, this may worsen BBB dysfunction and blood extravasation.

Along with an increase in ICP, CBF alterations are of clinical importance in acute recovery of severe TBI. Post-traumatic cerebral hypoperfusion is a common outcome and is also observed in the PBBI model (25, 31, 36). Theoretically, because of its efficacy in increasing cerebral perfusion pressure, REBOA may be an effective intervention toward augmenting CBF. However, neither cAO nor iAO were able to overcome the dramatic reductions in CBF induced by PBBI, possibly due to the vasculature damage and compromised blood pressure autoregulatory functions. These outcomes are common in severe TBI and may correspond to cerebral hypoperfusion (44–47). However, the decreased perfusion may not necessarily be detrimental to the injury pathology and immediate stabilization. Decreased CBF may reduce the total blood volume in the cranium and improve the ICP aberrations in accordance with the Monro-Kellie hypothesis. This correlation between ICP and CBF has been observed in TBI patients (48).

A potential complication of the limited blood flow is decreased metabolism and hypoxemia within the brain. This was observed as decreases in PbtO_2_ (Figure 6). Decreased PbtO_2_ and hypoxia are associated with poor outcomes in severe TBI patients (49). Furthermore, brain tissue oxygen-directed treatments have demonstrated clinical efficacy in improving outcomes (50). The results shown here demonstrate the ability of cAO to increase PbtO_2_ levels following PBBI. The effects of cAO on PbtO_2_ levels were most clearly seen during the occlusion, after which PbtO_2_ levels were comparable to those previously reported within the PBBI model (25). Further evidence for the exacerbated vascular damage following iAO may be provided by the lack of ability to elicit PbtO_2_ changes. No significant changes were observed in PbtO_2_ levels during intermittent occlusions. PbtO_2_ levels were decreased in the post-occlusion monitoring period compared to sham. These levels are also lower than previously reported values observed within the PBBI model (25).

Widespread implementation of REBOA is met with several challenges including insufficient clinical and preclinical data, and a lack of technological development (51, 52). The study described herein provides important pre-clinical data describing the relationship of aortic occlusion and cerebral physiology in an isolated penetrating TBI model, but there are important limitations that should be addressed. First, an isolated TBI model was used to determine the direct relationship between cerebral physiology of the injured brain and aortic occlusion. This model may be relevant to clinical cases of REBOA without hemorrhage (neurogenic shock, cerebral ischemia, and non-traumatic cardiac arrest), but REBOA is used predominantly for hemorrhage control and situations of hypovolemic shock. Future studies should employ polytrauma models to expand the understanding provided here. Another important limitation of the study results from the physiological differences between rodents and humans. While rodents offer an ideal testing platform for initial screening, these results should be replicated in a more biofidelic and gyrencephalic large animal model prior to clinical translation. In addition, normal and pathologic ranges for the measured outcomes of this study are more well-defined in humans as opposed to rats. As such, the data is expressed as percent change which more explicitly shows individualized changes throughout the experiment. Future studies will need to consider the magnitude of these changes along with the acceptable ranges. Finally, the acute physiological recovery evaluated here does not provide a complete evaluation of the effects on TBI and follow-up studies should provide an investigation of ongoing neuropathology.

## Conclusions

To our knowledge, this study provides the first look into the direct effects of aortic occlusion on cerebral physiology following TBI with severe intracranial hemorrhage and in the absence of a carotid rete mirabile. The results suggest that in the presence of TBI, cAO may improve cerebral perfusion with temporary improvements to ICP and PbtO_2_. However, iAO following TBI demonstrated adverse effects that may worsen clinical outcomes. This information should be incorporated into future pre-clinical studies in rodent polytrauma models that include hemorrhage. Ultimately, the goal of this work is to provide foundational knowledge of the relationship between aortic occlusion in the severely injured brain with the hope of enhancing the clinical implementation of REBOA in patients with concomitant TBI.

## Data Availability Statement

The datasets generated for this study are available on request to the corresponding author.

## Ethics Statement

The animal study was reviewed and approved by Institutional Animal Care and Use Committee of Walter Reed Army Institute of Research.

## Author Contributions

ZB performed data analysis, statistical analysis, and manuscript drafting. KC and XY performed experiments. JG, DS, TR, and LL performed experimental design and conception. LL oversaw experimentation and performed data analysis. All authors aided in the editing of the manuscript.

### Conflict of Interest

The authors declare that the research was conducted in the absence of any commercial or financial relationships that could be construed as a potential conflict of interest.
